# Effects of Prevalent and Incident Atrial Fibrillation on Renal Outcome, Cardiovascular Events, and Mortality in Patients with Chronic Kidney Disease

**DOI:** 10.3390/jcm8091378

**Published:** 2019-09-03

**Authors:** Hsin-Hui Hsu, Chew-Teng Kor, Yao-Peng Hsieh, Ping-Fang Chiu

**Affiliations:** 1 Department of Internal Medicine, Changhua Christian Hospital, Changhua 50006, Taiwan; 2 Division of Nephrology, Department of Internal Medicine, Changhua Christian Hospital, Changhua 50006, Taiwan; 3 School of Medicine, Kaohsiung Medical University, Kaohsiung 80708, Taiwan; 4 School of Medicine, Chung Shan Medical University, Taichung 40201, Taiwan; 5 Department of Recreation and Holistic Wellness, MingDao University, Changhua 52345, Taiwan

**Keywords:** chronic kidney disease (CKD), end-stage renal disease (ESRD), incident atrial fibrillation, mortality, stroke

## Abstract

Background: Little is known about how incident atrial fibrillation (AF) affects the clinical outcomes in chronic kidney disease (CKD) patients and whether there is a different influence between pre-existing and incident AF. Methods: Incident CKD patients from 2000 to 2013 were retrieved from the National Health Insurance Research Database (NHIRD) of Taiwan and they were classified as non-AF (*n* = 15,251), prevalent AF (*n* = 612), and incident AF (*n* = 588). The outcomes of interest were end-stage renal disease (ESRD) requiring dialysis, all-cause mortality, cardiovascular (CV) mortality, acute myocardial infarction (AMI), stroke or systemic thromboembolism. Results: Compared with CKD patients without AF, those with prevalent or incident AF were associated with higher adjusted rates of ESRD (hazard ratio (HR), 1.40; 95% confidence interval (CI), 1.32–1.48; HR, 2.91; 95% CI, 2.74–3.09, respectively), stroke or systemic thromboembolism (HR, 1.89; 95% CI, 1.77–2.03; HR, 1.67; 95% CI, 1.54–1.81, respectively), AMI (HR, 1.24; 95% CI, 1.09–1.41; HR, 1.99; 95% CI, 1.75–2.27, respectively), all-cause mortality (HR, 1.64; 95% CI, 1.56–1.72; HR, 2.17; 95% CI, 2.06–2.29, respectively), and CV mortality (HR, 2.95; 95% CI, 2.62–3.32; HR, 4.61; 95% CI, 4.09–5.20, respectively). Intriguingly, CKD patients with prevalent AF were associated with lower adjusted rates of ESRD, AMI, all-cause mortality, and CV mortality compared with those with incident AF. Conclusion: Both incident and prevalent AF were independently associated with greater risks of AMI, all-cause mortality, CV mortality, ESRD, and stroke or systemic thromboembolism. Our findings are novel in that, compared with prevalent AF, incident AF possessed an even higher risk of some clinical consequences, including ESRD, all-cause mortality, CV mortality, and AMI.

## 1. Introduction

Atrial fibrillation (AF) is the most common persistent cardiac arrhythmias and is estimated to affect 3 million people in the United States [1]. The changes in the atrial electrical properties to a diffuse and chaotic pattern that suppress normal sinus discharge initiate and perpetuate AF. AF not only causes many adverse cardiovascular consequences associated with reduced cardiac output and atrial thrombosis, including heart failure, strokes, and mortality, but also results in significant medical expenses [2,3,4]. The classification of AF is a matter of debate, but the most widely used system divides AF into paroxysmal, persistent, long-standing persistent, or permanent. All of these types require periodic monitoring and evaluation of thromboembolism risk. Beyond the established complication of AF-related thromboembolism in the brain and heart, AF has been reported to play a role in renal function deterioration among patients with chronic kidney disease (CKD) [5]. 

Similar to AF, the increase in the prevalence of CKD has trended globally and is mainly due to the growing aging population and the prevalence of diabetes mellitus (DM) and hypertension [6]. CKD is commonly complicated by a variety of comorbid conditions, which are contributors of adverse outcomes. In addition to the traditional risk factors, CKD itself, both reduced glomerular filtration rate and increased albuminuria, and CKD-specific milieu, such as anemia and mineral disorder, can also result in high cardiovascular morbidity and mortality risk [7]. The guidelines of the National Kidney Foundation and the American College of Cardiology/American Heart Association also consider CKD to be a risk equivalent to coronary heart disease [8,9]. CKD itself is also a causal risk factor for strokes independent of the traditional cardiovascular risk factor [10].

Several population-based studies have shown that CKD is a risk factor for new-onset AF [11,12,13]. The coexistence of CKD and AF can lead to a multiplicative risk rather than an additive risk from each entity alone. However, little is known about how incident AF affects the clinical outcomes in CKD patients and whether there is a different influence between pre-existing and incident AF. Thus, we conducted the present study using the nationwide healthcare claim data to investigate and compare the effects of incident AF and prevalent AF on mortality, end-stage renal disease (ESRD), cardiovascular (CV) mortality, acute myocardial infraction (AMI), and stroke or systemic thromboembolism in patients with predialysis CKD.

## 2. Experimental Section

### 2.1. Data Source

The Taiwan National Health Insurance Research Database (NHIRD) is large-scale, nationwide data and contains the comprehensive medical practices of >99% of Taiwan residents. The information was provided by contracted medical facilities to the Bureau of National Health Insurance (NHI) for claiming the reimbursement of medical expenses. We conducted a nationwide cohort study through retrieving the information of all patients with CKD from a subset of the Taiwan NHIRD. Our data of one million beneficiaries were randomly sampled from the original Taiwan NHIRD and released by the Bureau of NHI. There were no significant differences in age, gender, or healthcare expenditure between the sampled subset and the entire database. This study was evaluated and approved by our institutional review board. A waiver of informed consents was obtained due to the retrospective design and the encrypted identities of the enrollees. 

### 2.2. Study Cohort and Design

Taiwan NHIRD adopted the International Classification of Diseases, Ninth Revision, Clinical Modification (ICD-9-CM) to record the comorbidities. To increase the accuracy of diagnosis, we defined a patient to have a specific comorbidity if the medical diagnosis is listed as a discharge diagnosis or listed at least twice in the outpatient department within one year and the interval between the first and last date of medical coding should be more than 90 days apart [14,15]. First, we identified patients with CKD from 1 January 1996 and 31 December 2013 from the NHIRD [14,16]. Incident CKD patients from 2000 to 2013 were retrieved after excluding those CKD patients who had CKD diagnosed before 2000. This 4-year look-back period (1996–1999) was used to ensure that all CKD patients in our cohort were newly diagnosed and to reduce false incident cases. In addition, we also excluded those who were aged <18 or >100 years, underwent renal replacement therapy before the date of incident CKD, had incomplete demographic data, or a follow-up period of <90 days. The index date was defined as the date of first CKD confirmation. Finally, CKD patients with aortic or mitral valve disease or hyperthyroidism before the index date were also excluded. Individuals were classified as incident AF, prevalent AF, or non-AF according the temporal relations between the date of AF occurrence, if any, and the date of incident CKD. Patients with prevalent AF were those participants who already had AF before the diagnosis of CKD, whereas patients with incident AF were those who developed AF after the diagnosis of CKD, and the remaining patients were designated as non-AF. A follow-up started from the index date to the date of death, or the end of study on 31 December 2013. 

### 2.3. Study Outcomes and Relevant Confounding Variables

Our study outcomes were ESRD requiring renal replacement therapy, all-cause mortality, CV mortality, AMI, and stroke or systemic thromboembolism. Systemic thromboembolism included ischemic strokes, transient ischemic attacks, peripheral artery embolism, and pulmonary embolism. The events of AMI, stroke or systemic thromboembolism were defined as hospitalizations for those outcome events being listed as the first discharge diagnosis [17]. Diagnosis of ESRD requiring renal replacement therapy was confirmed by specific (ICD-9-CM) codes and inclusion in the Registry for Catastrophic Illness Patient Database, a sub-classification of the NHIRD. In Taiwan, ESRD patients undergoing renal replacement therapy were granted a catastrophic illness card so that the copayment for medical expenditure was waived. We also collected the data on pharmacotherapy, the frequency of annual outpatient visits, demographics, and comorbid conditions for statistical analyses. The CHA2DS2-VASc score, a valid stroke risk stratification model, was also calculated and adjusted for [18]. The medical codes used for the comorbidities, outcome events, and CHA2DS2-VASc calculation were provided in Table S1.

### 2.4. Statistical Analyses

The data distribution was shown in number (proportions) for categorical variables and in mean ± standard deviation (SD) for continuous variables in the three groups. The comparisons of the differences in the distribution of covariates in the three AF groups were made using one-way analysis of variance (ANOVA), a Chi-square test, or Fisher’s exact test, as appropriate. The crude incidence rate for each outcome of interest was calculated per every 1000 person-years for those with non-AF, prevalent AF, and incident AF, with associated 95% confidence interval (CI). The risk at study endpoints started when incident CKD was diagnosed. Traditional Kaplan–Meier curves are not suitable for our study because the incident AF group might have their AF occurrence years after their index CKD date. Instead, the Simon and Makuch method was adopted to plot the cumulative incidence of study outcomes for the three AF groups.

A propensity score-matched process has been widely applied to produce a similar distribution of baseline characteristics between case cohort and control cohort in observational studies examining the causal effects of treatments or interventions. Due to the three study groups, rather than two groups, in our study, we applied an inverse probability weighting (IPW) model with propensity scores estimated from the generalized boosted models for the concern over the distinct data distribution [19,20]. The generalized boosted model was a machine learning technique that applies an iterative process with multinomial regression tree to identify the propensity score model that produces the best covariate balance between exposure groups. All the baseline characteristics were used to calculate the propensity scores. 

A multivariate Cox regression model was built to analyze the association between study events and AF status by incorporating all the variables. Considering the competing risk of death and immortal bias for the incident AF group, we performed IPW-adjusted time-dependent cause-specific Cox models to examine the association between AF status and risk of study outcomes. Thus, if a patient developed AF during follow-up, they contributed time to the “non- AF” exposure group before being diagnosed with incident AF. After being diagnosed with AF, they would contribute person-time to the “incident AF” exposure group. The risk of study outcomes associated with AF status was expressed by hazard ratios (HRs) and 95% confidence interval (CI). The influence of AF status on study outcomes was examined by subgroup analyses stratified by age (< or ≥65 years), gender, CHA2DS2-VASc score (< or ≥3), and the use of renin-angiotensin system inhibitors. We performed three additional sensitivity analyses to test the robustness of our results. First, we repeated our analyses by incorporating those covariates with maximum standardization difference >0.1 into the adjustment model. Second, we adjusted for use of warfarin and antiplatelet agents (aspirin and clopidogrel) treated as time-dependent variables to determine whether treatment for AF attenuated the observed associations. Third, the risk of stroke or systemic thromboembolism was re-evaluated after excluding pulmonary embolism or a transient ischemic attack (TIA) as the outcome. Fourth, because the time difference in reporting AF is an important issue in our research and the misclassification of prevalent and incident AF is possible. We reclassified patients with AF occurring within 30, 90, and 180 days after CKD diagnosis to have prevalent AF to test the robustness of our study. All statistical analyses were performed using R language and SPSS statistical software, version 20.0 (SAS 9.4 software (SAS Institute Inc., Cary, NC, USA)). A two-tailed *p*-value <0.05 was considered statistically significant.

## 3. Results

### 3.1. Characteristics of Participants

**Figure 1:**
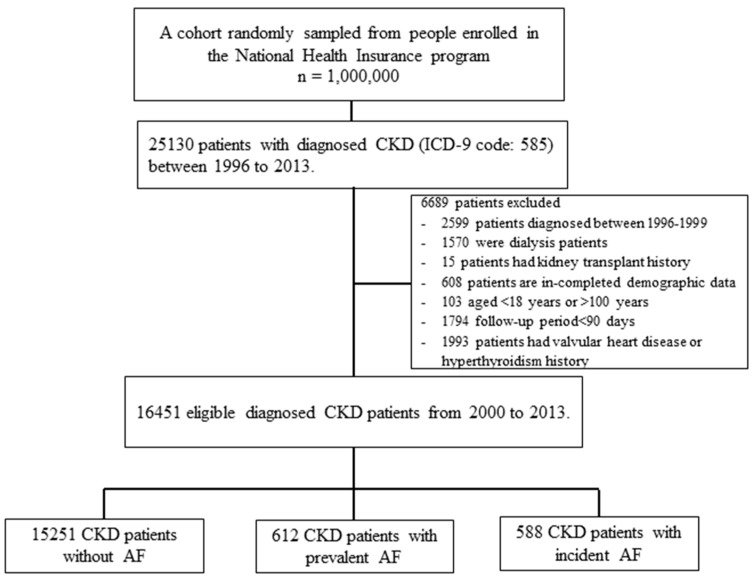
Flowchart of patient selection processes for incident chronic kidney disease (CKD) with non-atrial fibrillation (AF), prevalent AF, and incident AF.

The study data for the patient enrollment process were described in Figure 1. A total of 16,451 adults with incident CKD without valvular heart disease or hyperthyroidism were enrolled in this study and they were classified as the non-AF group (*n* = 15,251), prevalent AF group (*n* = 612), and incident AF group (*n* = 588). Before the IPW-matching process, participants with prevalent AF were more likely to be older, men, have a higher monthly income, have a past history of ischemic heart disease, chronic obstructive pulmonary disease, dementia, or peptic ulcer disease, and use more medications compared with the those with non-AF and incident AF (Table 1). After the IPW-matching process, no significantly different distribution amongst the three AF groups was found for most of the covariates, indicating their maximum standardization differences were less than 0.1.

### 3.2. Rates of Outcome Events by AF Status

**Figure 2:**
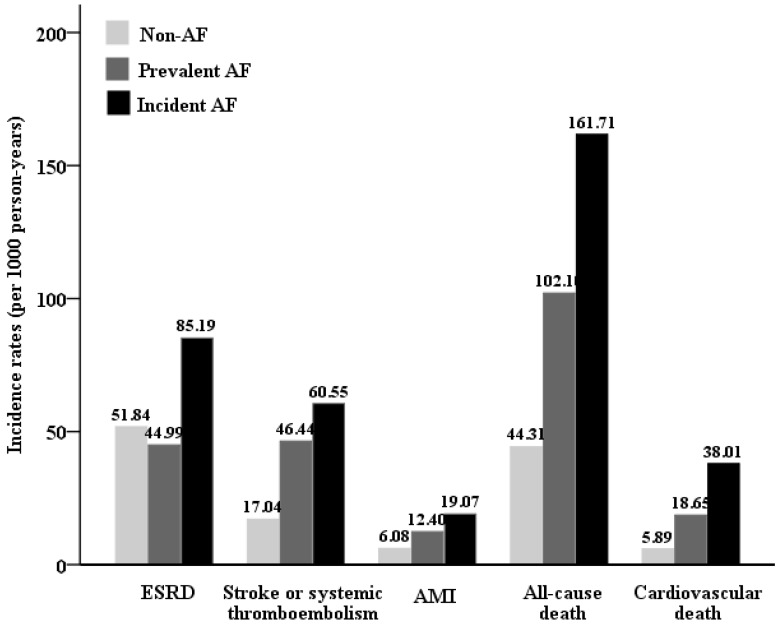
Cumulative incidence rates of study outcomes amongst participants with non-AF, prevalent AF, and incident AF.

The mean duration of follow-up was 4.72 ± 3.75 years. The crude incidence rate of our study outcomes was shown in Figure 2. The incident AF group had the highest incident rates for ESRD, stroke or systemic thromboembolism, AMI, all-cause mortality, and CV mortality compared with the prevalent and non-AF groups. Compared with non-AF group, the prevalent and incident AF groups both had several-fold higher crude HRs of ESRD (Table 2). Similarly, the prevalent and incident AF groups also had higher crude HRs of all-cause mortality, CV mortality, HF, AMI, ischemic stroke or systemic thromboembolism than the non-AF group. 

### 3.3. Association of AF Status with Subsequent ESRD 

The cumulative ESRD probability was plotted for the AF groups in Figure S1. In multivariable IPW-adjusted cause-specific and time-dependent Cox models (Table 2), incident AF was significantly associated with a 2.91-fold higher risk of ESRD, whereas prevalent AF was associated with a 1.4-fold higher risk compared with non-AF. Interestingly, incident AF had a significantly higher risk of ESRD compared with prevalent AF with the adjusted HR of prevalent AF versus incident AF being 0.48 (95% CI, 0.45–0.51). 

### 3.4. Association of AF Status with Subsequent All-Cause and CV Deaths

The cumulative incidence of all-cause and CV survival was plotted for the AF groups in Figures S2 and S3. In multivariable IPW-adjusted cause-specific and time-dependent Cox models (Table 2), incident AF was significantly associated with 2.17-fold and 4.61-fold higher risks of all-cause and CV mortality compared with non-AF, respectively. Prevalent AF was associated with 1.64-fold and 2.95-fold higher risks of all-cause and CV mortality compared with non-AF, respectively. Incident AF had a significantly higher risk of all-cause mortality and CV mortality compared with prevalent AF with the adjusted HR of prevalent AF versus incident AF for all-cause mortality and CV mortality being 0.76 (95% CI, 0.72–0.81) and 0.64 (95% CI, 0.57–0.72), respectively.

### 3.5. Association of AF Status with Subsequent Cardiovascular Events

The cumulative survival free of AMI and stroke or systemic thromboembolism was plotted for the AF groups in Figures S4 and S5. In multivariable IPW-adjusted cause-specific and time-dependent Cox models (Table 2), incident AF was significantly associated with higher adjusted rates of AMI (aHR, 1.99; 95% CI, 1.75–2.27) and stroke or systemic thromboembolism (aHR, 1.67; 95% CI, 1.54–1.81), whereas prevalent AF was also associated with higher adjusted rates of AMI (aHR, 1.24; 95% CI, 1.09–1.41) and stroke or systemic thromboembolism (aHR, 1.89; 95% CI, 1.77–2.03) compared with non-AF. Compared with incident AF, prevalent AF had a significantly higher adjusted HR of stroke or systemic thromboembolism (HR, 1.14; 95% CI, 1.05–1.25) but with a lower risk of AMI (HR, 0.62; 95% CI, 0.53–0.72).

### 3.6. Subgroup Analyses

The results of subgroup analyses stratified by age (< or ≥65 years), gender, CHA2DS2-VASc score (< or ≥3), and the use of an angiotensin-converting enzyme inhibitor/angiotensin II receptor blocker (ACEI/ARB) were shown in Table 3. The associations of increased risks of prevalent and incident AF versus non-AF with ESRD, AMI, all-cause mortality, CV mortality, and stroke or systemic thromboembolism were generally consistent across most of the participant subgroups. 

### 3.7. Sensitivity Analyses 

The associations of prevalent and incident AF versus non-AF with the outcomes under investigation remained statistically significant when the adjusted variables only included those with IPW-adjusted maximum standardization difference >0.1 in Table 1 (Table 4). In Cox models with medications (aspirin, clopidogrel, and warfarin) treated as time-dependent variables, very similar results to the main analyses were concluded regarding the associations of AF status with subsequent study outcomes. Additionally, consistent associations of prevalent and incident AF versus non-AF with subsequent risk of stroke or systemic thromboembolism were yielded when excluding TIA or pulmonary embolism as the outcome. Furthermore, the associations of a different AF status with clinical outcomes were consistent with preliminary results after reclassification of patients with AF occurring within 30, 90, and 180 days after CKD diagnosis as prevalent AF (Table S2).

## 4. Discussion

We analyzed well-characterized and large data from Taiwan NHIRD to study the impact of AF on the clinical endpoints in patients with CKD. To the best of our knowledge, the present study has been the first one to evaluate the differences in the risk of ESRD, AMI, mortality, and stroke or systemic thromboembolism in CKD patients stratified by the AF status. The main findings were highlighted as follows: (i) The presence of AF (prevalent or incident) was associated with higher risks of ESRD, all-cause mortality, CV mortality, AMI, and stroke or systemic thromboembolism compared with non-AF; (ii) the incident AF group had greater risks of ESRD, all-cause mortality, CV mortality, and AMI compared with the prevalent AF group.

AF-related ischemic strokes are a serious medical complication in CKD patients. Using Danish national registries, Olesen et al. also demonstrated a higher risk of ischemic strokes in patients with both CKD and AF and a reduced risk of thromboembolism by the warfarin treatment [21]. CKD per se was also suggested as a causal risk factor for strokes beyond traditional cardiovascular risk factors (10). CKD and AF share many cardiovascular risk factors, such as advanced age, hypertension, and coronary artery disease, so that the coexistence could lead to an amplified risk of a stroke. Bansal et al. examined the association of a new-onset of AF with subsequent risks of CV events in patients with CKD and found that participants with incident AF had a higher adjusted rate of strokes (HR, 2.66) compared with those without incident AF [22]. We also found the similar association, but the adjusted HR was 1.75. The difference in the strength of associations may be attributed to the distinct population characteristics, study design, and the number and severity of adjusted covariates. In addition to reporting the risk of thromboembolism for incident AF, we also compared the risk of prevalent AF and incident AF, which has rarely been discussed. Incident AF carried a lower risk of strokes versus prevalent AF from our study. 

The term “cardiorenal syndrome” refers to the acute or chronic dysfunction of the heart or kidney, which can induce acute or chronic dysfunction of the other organ [23,24]. Previous work has established AF to be a causal predictor for CKD [25]. Further studies also addressed the risk of ESRD associated with AF in adults with CKD. Bansal et al. reported a strong and independent association between incident AF and ESRD with an adjusted HR of 1.67 among CKD patients enrolled in the Kaiser Permanente Northern California study [26]. The authors further used a more vigorous prospective design to study 3939 participants in the Chronic Renal Insufficiency Cohort (CRIC) Study and found that incident AF was associated an even higher risk of ESRD (adjusted HR 3.2) [22]. Consistent with their later study, we also showed that incident AF was associated with a nearly three-fold higher risk of ESRD compared with non-AF. Moreover, incident AF even carried a higher risk of ESRD than prevalent AF. 

AF has been associated with higher risks of AMI, heart failure, and death in individuals without CKD [27,28,29,30]. In a meta-analysis of 104 cohort studies involving 9.7 million participants, AF was associated with a 1.46- to 4.99-fold higher risk of death, heart failure, and ischemic heart disease [31]. AF also carried a higher risk of death in patients with ESRD who underwent hemodialysis [32,33]. Research on the impact of incident AF on those outcomes in patients with CKD is relatively scarce. In a study of CKD patients in a large healthcare service system, incident AF was associated with a 66% increase in the relative rate of death (adjusted HR 1.66, 95% CI 1.57 to 1.77) [34]. Recently, one population-based retrospective cohort study of 1.4 million adult residents with eGFRs < 90 mL/min/1.73 m^2^ from Ontario, Canada found that incident AF is associated with a high risk for adverse outcomes, including HF, ESRD, death, and AMI, particularly in the first six months from diagnosis [35]. Later, a prospective cohort study of 3939 adults enrolled into the CRIC study by Bansal et al. reported that incident AF is independently associated with two- to five-fold increased rates of developing subsequent heart failure, myocardial infarction, strokes, or death in adults with CKD [22]. Similar to their findings, we also found that incident AF and prevalent AF were associated with higher risks of mortality and AMI compared with non-AF. Although retrospectively designed, our study had a much larger CKD population than Bansal’s. Furthermore, the results of our study can be applicable to CKD patients in the Asia-Pacific region amid most studies regarding the influence of incident AF on clinical outcomes that have been conducted in Europe or the United States. 

There are several possibilities to explain our findings. AF and CKD share many pathogenic mechanisms, making them closely interrelated. The increased fibroblast growth factor-23 level in CKD has been linked with left ventricular hypertrophy and increased left ventricular mass, resulting in left ventricular dysfunction [36]. AF-related tachycardia can cause an inadequate cardiac filling volume and it can also lead to structural and functional cardiac damage in the long run. Vascular calcification in CKD and reduced coronary perfusion by AF can contribute to insufficient blood supply to the coronary arteries. Briefly, AF and CKD amplify a vicious cycle, resulting in higher overall death and CV death. Furthermore, atrial thrombi may migrate to cause coronary and cerebral embolism as a possible mechanism of AMI and ischemic strokes. Therefore, AF undoubtedly confers a significant association with adverse cardiovascular complications. AF-induced alteration in cardiac hemodynamics can promote the progression of CKD to ESRD via reduced renal perfusion [37]. Other possible mechanisms of renal function deterioration by AF include direct impaired renal hemodynamics, increased fibrosis within myocardium and renal tissues, up-regulation of pro-fibrotic proteins, and possible renal microinfarctions [38,39]. 

The present study also had some limitations. First, the diagnosis of AF was made based on the diagnostic code registered by the attending physician and was not verified by the electrocardiogram, which is not available in the NHIRD. It is possible that paroxysmal or asymptomatic AF may be undiagnosed in the non-AF group amid electrocardiogram and cardiac event recording are not a routine for CKD care. Since AF leads to a higher risk of adverse clinical outcomes, the association of AF with study events will be strengthened more after re-classifying those with paroxysmal AF in the non-AF group as AF group. Moreover, we adjusted for the frequency of medical visits to mitigate detection bias since AF occurrence could be more easily detected in patients with more medical utilization. Second, some important factors known to affect AF and study outcomes were not available in the national registry data and included smoking habits, alcohol consumption, and physical activities. Nevertheless, we adjusted for coronary artery disease, COPD, and liver cirrhosis, which are the surrogate markers of unhealthy lifestyle habits. Third, we also lacked echocardiographic parameters and thyroid function tests. We excluded participants with aortic, mitral valve disorders or hyperthyroidism at study entry because valvular heart disease and thyroid disorder can predispose to AF occurrence and mediate the observed association. Finally, most patients with AF were not treated with oral anticoagulation. However, the society guideline for stroke prophylaxis in AF patients has changed a lot with time. In addition, the use of antiplatelet or anticoagulant relies on the clinical guidelines at the time, the patient’s preference, and the physician’s experience. Thus, our data on medication use represents a real-world setting. Although the use of warfarin in the prevalent AF group was only 12%, the use of anti-platelet drugs was nearly 60%. The total rate of using antiplatelet or anticoagulant drugs achieved more than 70%. 

## 5. Conclusions

In conclusion, both incident and prevalent AF were independently associated with greater risks of AMI, all-cause mortality, CV mortality, ESRD, and stroke or systemic thromboembolism in a large adult population with CKD. Our findings are novel in that, compared with prevalent AF, incident AF possessed an even higher risk of some clinical consequences, including ESRD, death, and AMI. Therefore, cardiac rhythms should be monitored periodically amongst CKD patients as a routine practice in the integrated CKD care program in the hope of early detection of incident AF. However, the underlying mechanisms of why incident AF confers to higher risks of some adverse outcomes than prevalent AF are beyond the scope of this study due to its retrospective nature, and further studies to delineate the pathogenesis are mandatory for the aim of exploiting evidence-based practice and putting into practice targeted at reducing the renal, cardiovascular, and thrombotic events.

## Figures and Tables

**Table 1:** Baseline characteristics of study population between the AF groups.

	CKD Cohort				Maximum Standardization Difference between Groups	
	Non-AF	Prevalent AF	Incident AF	*p*-Value	Before IPW ^a^ (%)	After IPW ^a^ (%)
Sample size	15,251	612	588	--	--	--
Age, years	65 ± 14	76 ± 10	72 ± 10	<0.001	0.746	0.161
Gender, Male	9076 (59.51%)	397 (64.87%)	337 (57.31%)	0.015	0.111	0.098
Monthly income, NTD	14,144.97 ± 13,893.61	10,331.85 ± 11,240.98	10,067.77 ± 10,329.03	<0.001	0.340	0.119
Geographical location						
Northern	6509 (42.68%)	264 (43.14%)	246 (41.84%)	0.894	0.028	0.093
Central	2799 (18.35%)	112 (18.3%)	121 (20.58%)	0.392	0.073	0.088
Southern	5521 (36.2%)	207 (33.82%)	197 (33.5%)	0.211	0.073	0.035
Eastern	422 (2.77%)	29 (4.74%)	24 (4.08%)	0.004	0.094	0.042
Comorbidities						
Coronary artery disease	2881 (18.89%)	254 (41.5%)	197 (33.5%)	<0.001	0.559	0.127
Chronic obstructive pulmonary disease	1451 (9.51%)	137 (22.39%)	99 (16.84%)	<0.001	0.424	0.081
Cancer	1119 (7.34%)	50 (8.17%)	32 (5.44%)	0.156	0.116	0.021
Liver Cirrhosis	361 (2.37%)	16 (2.61%)	5 (0.85%)	0.050	0.117	0.140
Dementia	379 (2.49%)	30 (4.9%)	16 (2.72%)	0.001	0.152	0.113
Rheumatoid disease	317 (2.08%)	14 (2.29%)	9 (1.53%)	0.609	0.040	0.021
Peptic ulcer disease	2934 (19.24%)	151 (24.67%)	127 (21.6%)	0.002	0.137	0.046
CHA2DS2-VASc score	3 ± 2	4 ± 2	3 ± 1	<0.001	0.806	0.102
0	1159 (7.6%)	8 (1.31%)	8 (1.36%)	<0.001		
1	2903 (19.03%)	35 (5.72%)	40 (6.8%)	<0.001		
2	3674 (24.09%)	79 (12.91%)	124 (21.09%)	<0.001		
3	3360 (22.03%)	141 (23.04%)	152 (25.85%)	0.081		
4	2317 (15.19%)	140 (22.88%)	135 (22.96%)	<0.001		
5	1200 (7.87%)	109 (17.81%)	81 (13.78%)	<0.001		
≥6	638 (4.18%)	100 (16.34%)	48 (8.16%)	<0.001		
Long-term medication use						
ACEI/ARB	6537 (42.86%)	383 (62.58%)	288 (48.98%)	<0.001	0.396	0.074
beta-blocker	4948 (32.44%)	324 (52.94%)	237 (40.31%)	<0.001	0.432	0.125
Diuretics	4192 (27.49%)	328 (53.59%)	233 (39.63%)	<0.001	0.573	0.083
Statin	4133 (27.1%)	185 (30.23%)	139 (23.64%)	0.037	0.194	0.117
NSAIDs	2286 (14.99%)	131 (21.41%)	107 (18.2%)	<0.001	0.179	0.012
Pentoxifylline	1028 (6.74%)	63 (10.29%)	50 (8.5%)	0.001	0.139	0.114
ESA	130 (0.85%)	3 (0.49%)	3 (0.51%)	0.430	0.047	0.090
Aspirin/clopidogrel	3929 (25.76%)	362 (59.15%)	203 (34.52%)	<0.001	0.748	0.105
Warfarin	141 (0.92%)	76 (12.42%)	8 (1.36%)	<0.001	0.99	0.029
Annual frequency of medical visits	30 ± 20	34 ± 20	34 ± 22	<0.001	0.218	0.080

Values are expressed as mean ± SD or number (%). ^a^ Inverse probability of group-weighting (IPW) was estimated by the propensity of group from generalized boosted regression. Chronic kidney disease (CKD); atrial fibrillation (AF); angiotensin-converting enzyme inhibitor (ACEI); angiotensin II receptor blocker (ARB); New Taiwan Dollar (NTD); Non-Steroidal Anti-Inflammatory Drug (NSAID); erythropoiesis-stimulating agents (ESA).

**Table 2:** Risks for end-stage renal disease (ESRD), stroke or systemic thromboembolism, acute myocardial infarction (AMI), and mortality among patients with CKD by AF status.

Outcome	Event	IR (95% CI)	Weighted Time-Dependent Cox Model					
			cHR (95% CI)	*p*-Value	aHR (95% CI)	*p*-value	aHR (95% CI)	*p*-Value
ESRD								
Non-AF	3440	51.84 (50.11–53.57)	1		1		0.34 (0.32–0.36)	<0.0001
Prevalent AF	89	44.99 (35.64–54.34)	1.29 (1.22–1.36)	<0.001	1.40 (1.32–1.48)	<0.001	0.48 (0.45–0.51)	<0.0001
Incident AF	95	85.19 (68.06–102.32)	3.05 (2.88–3.23)	<0.001	2.91 (2.74–3.09)	<0.001	1	
Stroke or systemic thromboembolism								
Non-AF	1247	17.04 (16.09–17.98)	1		1		0.61 (0.56–0.66)	<0.0001
Prevalent AF	89	46.44 (36.79–56.09)	2.02 (1.88–2.16)	<0.001	1.89 (1.77–2.03)	<0.001	1.14 (1.05–1.25)	0.002
Incident AF	79	60.55 (47.19–73.9)	2.51 (2.33–2.72)	<0.001	1.67 (1.54–1.81)	<0.001	1	
Acute myocardial infarction								
Non-AF	461	6.08 (5.52–6.63)	1		1		0.50 (0.44–0.57)	<0.0001
Prevalent AF	26	12.4 (7.64–17.17)	1.34 (1.18–1.52)	<0.001	1.24 (1.09–1.41)	0.001	0.62 (0.53–0.72)	<0.0001
Incident AF	28	19.07 (12.01–26.13)	3.02 (2.67–3.41)	<0.001	1.99 (1.75–2.27)	<0.001	1	
All-cause mortality								
Non-AF	3399	44.31 (42.82–45.8)	1		1		0.46 (0.44–0.49)	<0.0001
Prevalent AF	219	102.1 (88.58–115.62)	1.83 (1.74–1.92)	<0.001	1.64 (1.56–1.72)	<0.001	0.76 (0.72–0.81)	<0.0001
Incident AF	251	161.71 (141.7–181.72)	3.11 (2.96–3.27)	<0.001	2.17 (2.06–2.29)	<0.001	1	
Cardiovascular mortality								
Non-AF	452	5.89 (5.35–6.44)	1		1		0.22 (0.19–0.25)	<0.0001
Prevalent AF	40	18.65 (12.87–24.43)	3.18 (2.83–3.57)	<0.001	2.95 (2.62–3.32)	<0.001	0.64 (0.57–0.72)	<0.0001
Incident AF	59	38.01 (28.31–47.71)	6.81 (6.08–7.63)	<0.001	4.61 (4.09–5.20)	<0.001	1	

Confidence interval (CI); hazard ratio (HR); incidence rate (IR; per 1000 person-years); end-stage renal disease (ESRD); acute myocardial infarction (AMI); chronic kidney disease (CKD); atrial fibrillation (F). aHR was calculated from adjustment for all variables in Table 1.

**Table 3:** Adjusted associations of AF status with risk of clinical outcomes stratified by sex, age, CHA2DS2-VASc score, and medication use.

Subgroup	ESRD		Stroke or Systemic Thromboembolism		AMI		All-Cause Mortality		Cardiovascular Mortality	
	Prevalent AF vs. Non-AF	Incident AF vs. Non-AF	Prevalent AF vs. Non-AF	Incident AF vs. Non-AF	Prevalent AF vs. Non-AF	Incident AF vs. Non-AF	Prevalent AF vs. Non-AF	Incident AF vs. Non-AF	Prevalent AF vs. Non-AF	Incident AF vs. Non-AF
***Age***										
Age < 65	1.48 (1.36–1.62)	3.02 (2.75–3.31)	1.93 (1.75–2.13)	1.67 (1.52–1.83)	1.68 (1.49–1.89)	2.18 (1.97–2.42)	1.98 (1.71–2.29)	2.07 (1.83–2.33)	3.27 (2.47–4.33)	6.92 (5.01–9.56)
Age ≥ 65	1.30 (1.20–1.41)	2.86 (2.63–3.11)	1.88 (1.72–2.05)	1.7 (1.53–1.89)	1.11 (0.95–1.30)	1.47 (1.23–1.77)	1.78 (1.68–1.88)	2.25 (2.12–2.40)	2.92 (2.55–3.35)	5.20 (4.51–5.98)
*p* for interaction	0.029	<0.001	0.104	0.650	0.037	<0.001	<0.001	0.529	0.436	0.610
***Gender***										
Female	1.48 (1.36–1.61)	3.27 (2.99–3.57)	1.66 (1.45–1.89)	2.15 (1.89–2.44)	0.98 (0.76–1.26)	2.20 (1.75–2.77)	1.56 (1.44–1.70)	2.72 (2.51–2.96)	3.64 (3.05–4.35)	8.28 (6.95–9.87)
Male	1.27 (1.18–1.38)	2.92 (2.68–3.17)	2.08 (1.90–2.26)	1.34 (1.20–1.49)	1.43 (1.22–1.66)	1.89 (1.61–2.21)	1.69 (1.58–1.80)	1.93 (1.8–2.07)	2.70 (2.28–3.20)	2.96 (2.47–3.55)
*p* for interaction	0.005	0.119	<0.001	<0.001	0.013	0.012	0.430	<0.001	0.004	<0.001
***CHA2DS2-VASc Score***										
CHA2DS2-VASc Score ≤3	1.65 (1.54–1.77)	3.37 (3.13–3.62)	2.40 (2.18–2.64)	2.31 (2.09–2.55)	1.92 (1.62–2.28)	3.52 (2.98–4.14)	1.96 (1.83–2.10)	2.13 (1.99–2.28)	3.17 (2.67–3.77)	5.78 (4.87–6.85)
CHA2DS2-VASc Score >3	1.02 (0.92–1.13)	3.23 (2.9–3.59)	1.54 1.38–1.72)	2.04 (1.79–2.34)	1.04 (0.60–1.78)	2.34 (1.89–2.89)	1.44 (1.33–1.56)	3.10 (2.87–3.35)	1.56 (1.28–1.89)	8.41 (7.12–9.93)
*p* for interaction	<0.001	0.408	<0.001	0.001	<0.001	<0.001	<0.001	<0.001	<0.001	<0.001
***Renin-angiotensin system inhibitors use***										
Non-user	1.47 (1.34–1.6)	2.46 (2.23–2.71)	1.99 (1.87–2.11)	2.03 (1.9–2.18)	1.58 (1.47–1.69)	2.68 (2.5–2.88)	1.68 (1.57–1.81)	2.57 (2.39–2.77)	4.79 (3.99–5.76)	4.97 (4.08–6.06)
User	1.44 (1.33–1.56)	3.52 (3.24–3.81)	1.29 (1.16–1.43)	2.09 (1.88–2.33)	1.18 (0.74–1.87)	1.03 (0.54–1.98)	1.76 (1.63–1.89)	2.02 (1.87–2.18)	2.07 (1.75–2.46)	5.27 (4.51–6.16)
*p* for interaction	0.909	<0.001	<0.001	<0.001	0.7437	<0.001	0.4018	0.3070	<0.001	0.847

Atrial fibrillation (AF); angiotensin-converting enzyme inhibitor (ACEI); angiotensin II receptor blocker (ARB); end-stage renal disease (ESRD); acute myocardial infarction (AMI).

**Table 4:** Sensitivity analyses.

Outcome	Weighted Time-Dependent Cox Model					
	aHR ^a^ (95% CI)	*p*-Value	aHR ^b^ (95% CI)	*p*-Value	aHR ^c^ (95% CI)	*p*-Value
**ESRD**						
Non-AF	1		1			
Prevalent AF	1.41 (1.33–1.49)	<0.001	1.44 (1.36–1.52)	<0.0001		
Incident AF	2.85 (2.68–3.02)	<0.001	2.90 (2.73–3.08)	<0.0001		
**Stroke**						
Non-AF	1		1			
Prevalent AF	1.90 (1.77–2.04)	<0.001	1.88 (1.75–2.02)	<0.0001		
Incident AF	1.86 (1.72–2.02)	<0.001	1.82 (1.67–1.97)	<0.0001		
**Acute myocardial infarction**						
Non-AF	1		1			
Prevalent AF	1.27 (1.11–1.44)	<0.001	1.2 (1.05–1.36)	0.005		
Incident AF	2.22 (1.95–2.53)	<0.001	2.09 (1.84–2.38)	<0.0001		
**All-cause mortality**						
Non-AF	1		1			
Prevalent AF	1.71 (1.63–1.80)	<0.001	1.72 (1.63–1.80)	<0.0001		
Incident AF	2.51 (2.38–2.65)	<0.001	2.49 (2.36–2.62)	<0.0001		
**CV mortality**						
Non-AF	1		1			
Prevalent AF	2.88 (2.56–3.23)	<0.001	3.29 (2.91–3.71)	<0.0001		
Incident AF	5.08 (4.51–5.72)	<0.001	4.6 2(4.10–5.21)	<0.0001		
**Stroke (excluding TIA)**						
Non-AF	1		1		1	
Prevalent AF	2.02 (1.88–2.18)	<0.0001	1.82 (1.69–1.96)	<0.0001	2.02 (1.88–2.17)	<0.0001
Incident AF	1.87 (1.72–2.03)	<0.0001	1.49 (1.36–1.62)	<0.0001	1.66 (1.53–1.81)	<0.0001
**Stroke (excluding pulmonary embolism)**						
Non-AF	1		1	1	1	
Prevalent AF	2.10 (1.95–2.26)	<0.0001	1.87 (1.73–2.02)	2.10 (1.95–2.26)	2.05 (1.91–2.21)	<0.0001
Incident AF	1.95 (1.79–2.13)	<0.0001	1.60 (1.46–1.75)	1.95 (1.79–2.13)	1.78 (1.63–1.94)	<0.0001

^a^ Adjusted for those variables with a maximum standardization difference >0.10; i.e., age, monthly income, CHA2DS2-VASc score, ischemic heart disease, liver cirrhosis, dementia, beta-blockers, statin, pentoxyfilline, and aspirin/clopidogrel. ^b^ Adjusted for all the variables in Table 1 with medications (aspirin/clopidogrel and warfarin) treated as time-dependent variables. ^c^ Adjusted for all the variables in Table 1. Transient ischemic attack (TIA).

## Supplementary Materials

The following are available online at https://www.mdpi.com/2077-0383/8/9/1378/s1, Figure S1: Cumulative incidence of ESRD amongst participants with non-AF, prevalent AF and incident AF. Figure S2: Cumulative survival curves amongst participants with non-AF, prevalent AF and incident AF. Figure S3: Cumulative survival curves free of cardiovascular death amongst participants with non-AF, prevalent AF and incident AF. Figure S4: Cumulative survival curves free of stroke of systemic thromboembolism amongst participants with non-AF, prevalent AF and incident AF. Figure S5: Cumulative survival curves free of acute myocardial infarction amongst participants with non-AF, prevalent AF and incident AF. Table S1: ICD-9-CM codes used to identify chronic kidney disease, atrial fibrillation, comorbidities and the cause of death. Table S2: Risks for ESRD, stroke or systemic thromboembolism, acute myocardial infarction and mortality among patients with CKD by AF status after re-classification of incident AF as prevalent AF (non-AF as the reference).
