# Supplementary material for: Effects of Prevalent and Incident Atrial Fibrillation on Renal Outcome, Cardiovascular Events, and Mortality in Patients with Chronic Kidney Disease
Source: J Clin Med. 2019 Sep 3;8(9):1378. doi: 10.3390/jcm8091378 (PMC6780958; doi:10.3390/jcm8091378)
Supplement: Supplementary file 1 [file jcm-08-01378-s001.pdf]

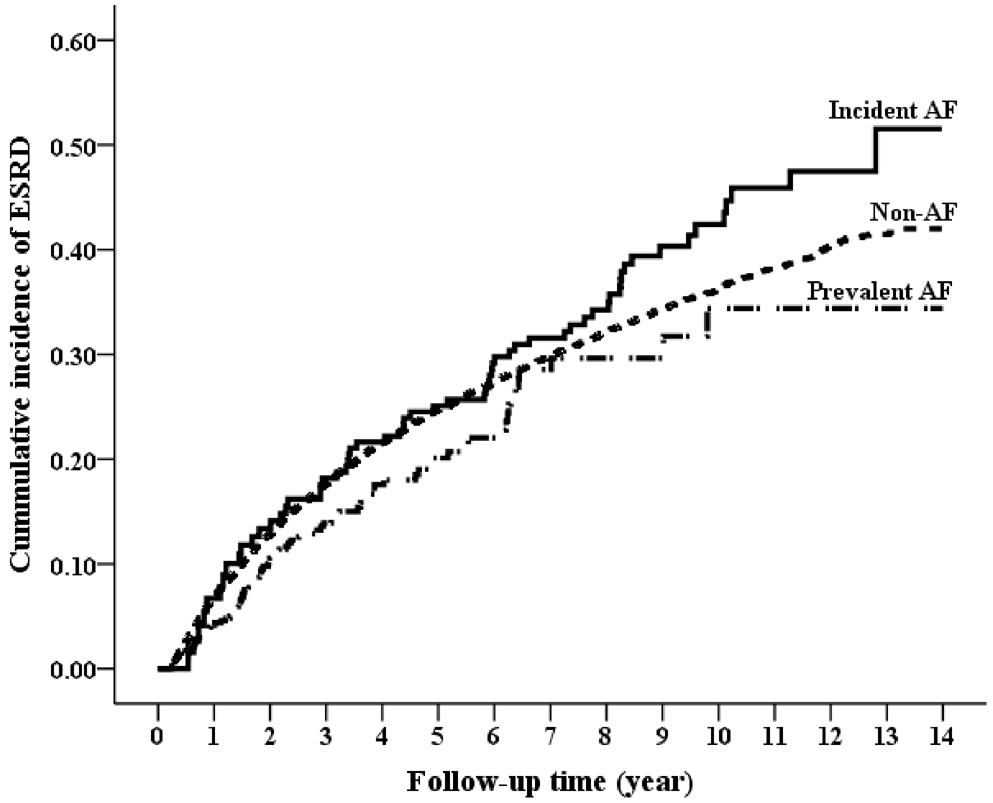

Figure 1. Cumulative incidence of ESRD amongst participants with non-AF, prevalent AF and incident AF.

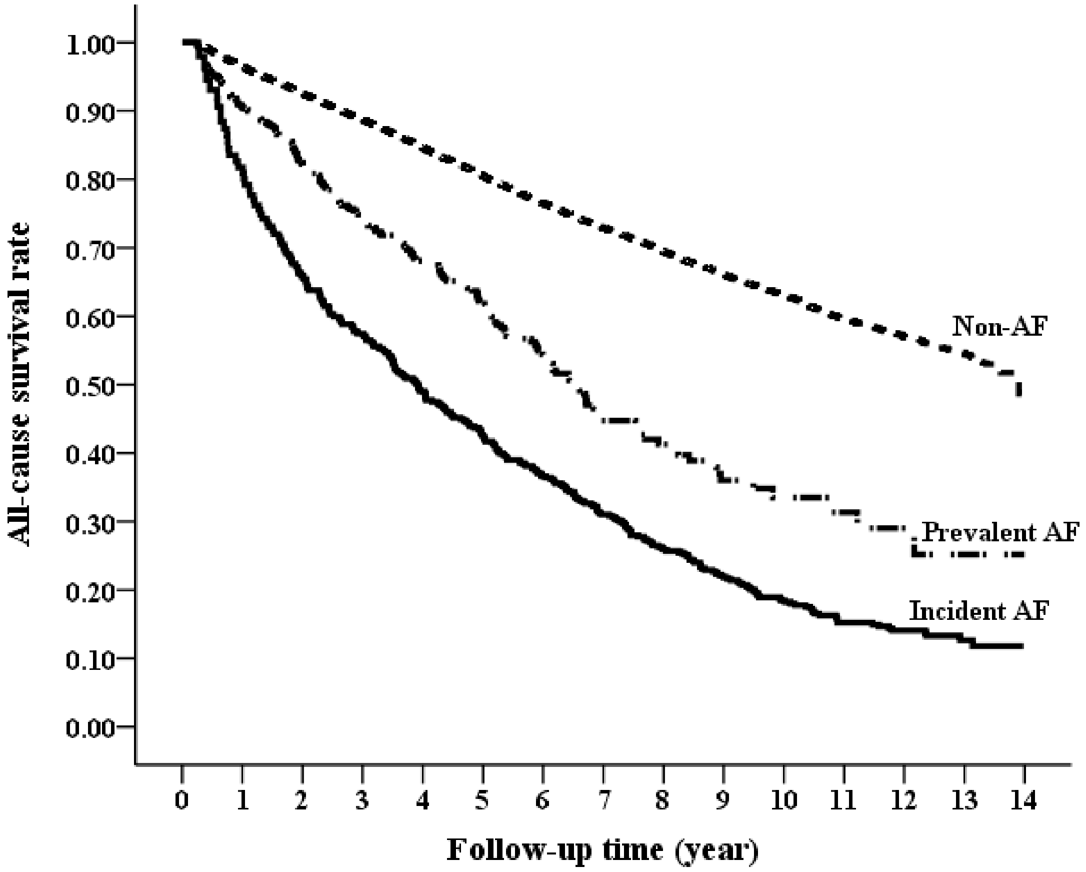

Figure S2: Cumulative survival curves amongst participants with non-AF, prevalent AF and incident AF.

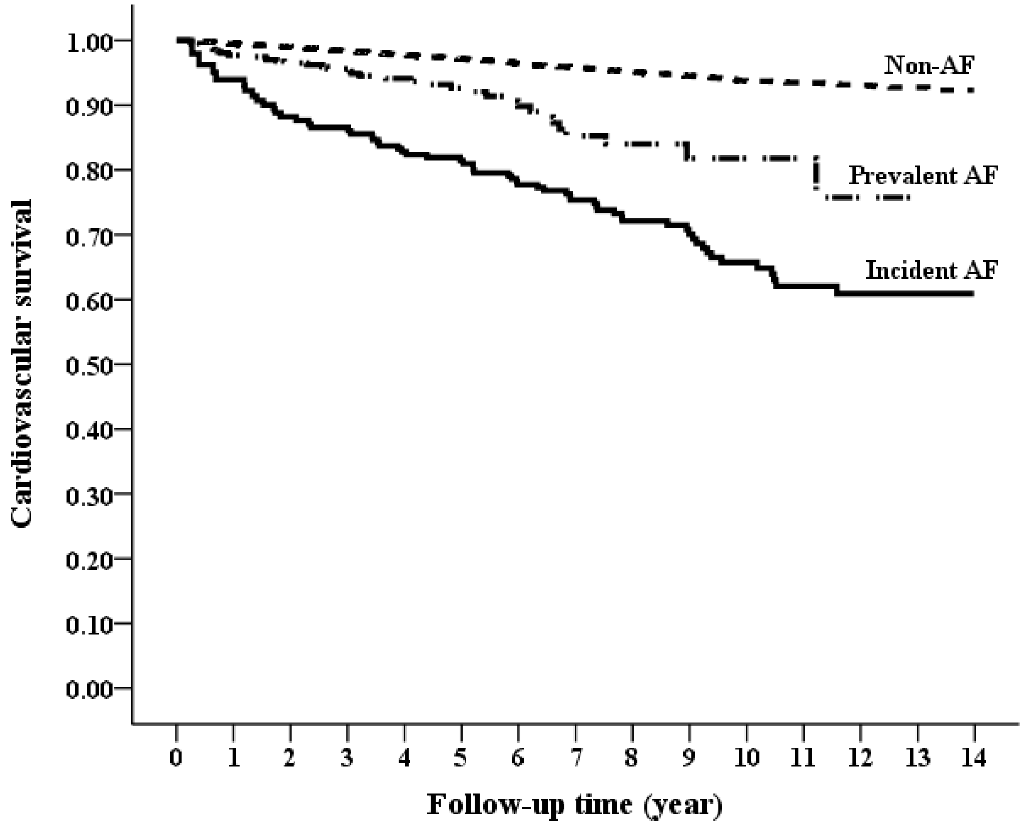

Figure S3: Cumulative survival curves free of cardiovascular death amongst participants with non-AF, prevalent AF and incident AF.

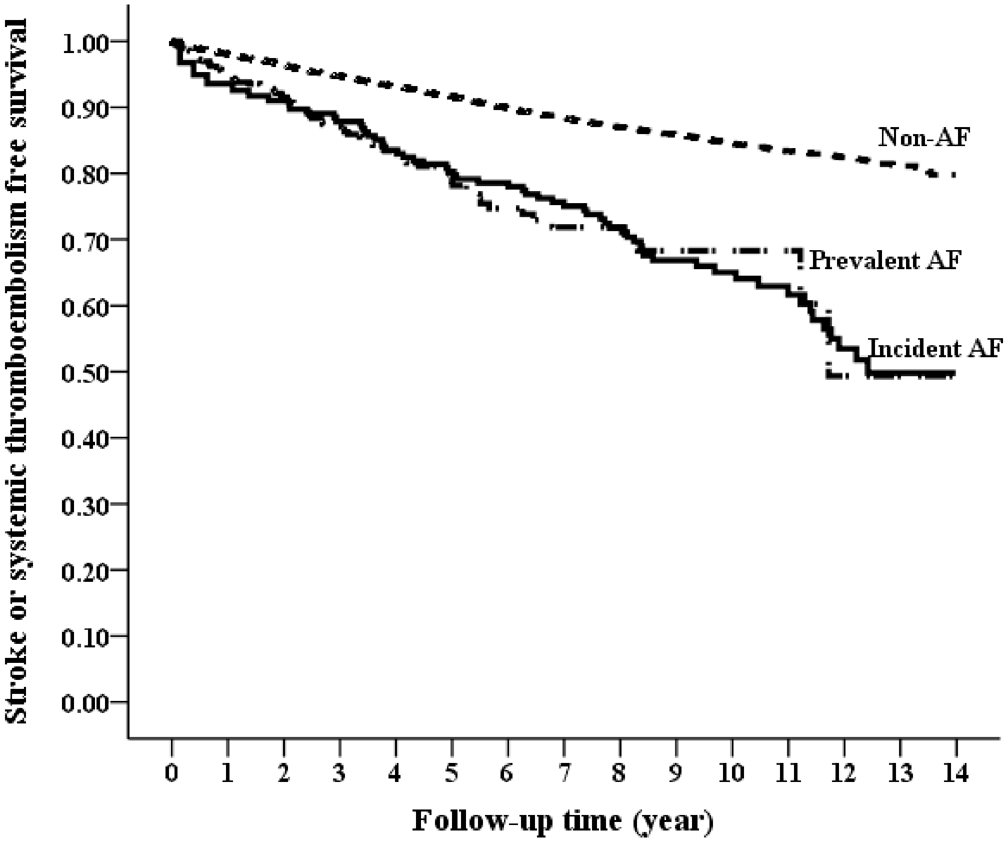

Figure S4: Cumulative survival curves free of stroke of systemic thromboembolism amongst participants with non-AF, prevalent AF and incident AF.

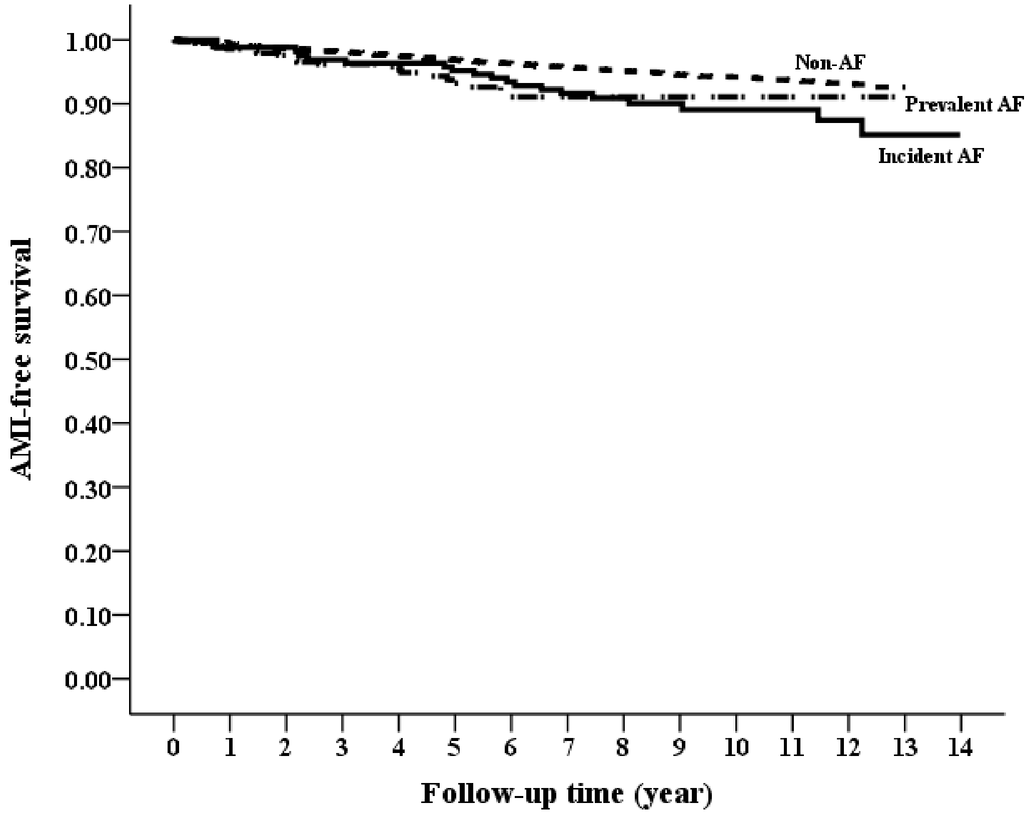

Figure S5: Cumulative survival curves free of acute myocardial infarction amongst participants with non-AF, prevalent AF and incident AF.

Supplementary Table 1. ICD-9-CM codes used to identify chronic kidney disease, atrial fibrillation, comorbidities and the cause of death.

Diseases	Corresponding ICD-9-CM codes
Chronic kidney disease	585.x
Atrial fibrillation	427.31
Acute myocardial infarction	410, 429.5, 429.6, 429.71, 429.79
Co-morbid diseases	
Coronary artery disease	410.x–414.x
COPD	491.x, 492.x, 496.x
Cancer	140-165, 170-172, 174-195, 200-208
Cirrhosis	571
Dementia	290
Rheumatoid disease	446.5, 710, 714, 725
Peptic ulcer disease	531-534
CHA2DS2-VASc	

Heart failure	428.x
Hypertension	401.x–405.x
Diabetes mellitus	250.x
Ischemic stroke	433, 434
Transient ischemic attack	435.9, 435.2, 435.8
Peripheral artery embolism	444
Pulmonary embolism	415.0, 415.9
Acute myocardial infarction	410, 429.5, 429.6, 429.71, 429.79
Aortic plaque	440.0, 440.2, 440.30, 440.31, 440.32, 440.71, 440.8, 440.9, 441, 443.9, 443.91
**Causes of Death**	
Cardiovascular death	390.x – 398.x, 410.x – 414.x, 4151, 41511, 41519, 420.x, 422.x – 429.x, 431.x, 433.x – 436.x, 518.4, 785.51

Abbreviation: COPD, chronic obstructive pulmonary disease; ICD-9-CM, International Classification of Disease, 9th Revision, Clinical Modification.

Supplementary Table 2. Risks for ESRD, stroke or systemic thromboembolism, acute myocardial infarction and mortality among patients with CKD by AF status after re-classification of incident AF as prevalent AF (non-AF as the reference)

	Within 30 days		Within 90 days		Within 180 days	
	aHR (95% CI)	P-value	aHR (95% CI)	P-value	aHR (95% CI)	P-value
**ESRD**						
Non-AF	1		1		1	
Prevalent AF	1.47(1.39,1.55)	<.0001	1.41(1.34,1.50)	<.0001	1.62(1.53,1.71)	<.0001
Incident AF	3.27(3.08,3.48)	<.0001	3.73(3.50,3.97)	<.0001	3.24(3.03,3.47)	<.0001
**Stroke**						
Non-AF	1		1		1	
Prevalent AF	1.75(1.63,1.88)	<.0001	1.77(1.65,1.90)	<.0001	1.76(1.64,1.89)	<.0001
Incident AF	1.49(1.37,1.62)	<.0001	1.46(1.34,1.59)	<.0001	1.61(1.47,1.76)	<.0001
**Acute myocardial infarction**						
Non-AF	1		1		1	
Prevalent AF	1.08(0.95,1.23)	0.260	1.01(0.89,1.15)	0.8473	1.01(0.89,1.15)	0.892
Incident AF	1.75(1.54,2.00)	<.0001	1.95(1.70,2.23)	<.0001	2.16(1.88,2.48)	<.0001
**All-cause mortality**						
Non-AF	1		1		1	
Prevalent AF	1.70(1.62,1.79)	<.0001	1.78(1.70,1.87)	<.0001	1.77(1.69,1.86)	<.0001
Incident AF	2.29(2.17,2.41)	<.0001	2.22(2.10,2.34)	<.0001	2.30(2.17,2.43)	<.0001
**CV mortality**						
Non-AF	1		1		1	
Prevalent AF	3.22(2.85,3.63)	<.0001	3.46(3.08,3.89)	<.0001	3.38(3.01,3.79)	<.0001

Incident AF	4.81(4.25,5.43)	<.0001	4.75(4.19,5.39)	<.0001	4.98(4.38,5.67)	<.0001

CI, confidence interval; HR, hazard ratio; CKD, chronic kidney disease; AF, atrial fibrillation.

aHR was calculated from adjustment for all variables in Table 1 with medications (aspirin / clopidogrel and warfarin) treated as time-dependent variables
